# Supplementary material for: Morphological and mechanical characterization of bone phenotypes in the Amish G610C murine model of osteogenesis imperfecta
Source: PLoS One. 2021 Aug 27;16(8):e0255315. doi: 10.1371/journal.pone.0255315 (PMC8396767; doi:10.1371/journal.pone.0255315)
Supplement: S1 Table — Significant effects (p>0.05) are highlighted in gray. Significant post-hoc values (p>0.05) between genotypes are marked with *, and between sex are marked with $. Data presented as mean +/- standard deviation. (DOCX) [file pone.0255315.s001.docx]

**Vertebral µCT Methods**

To analyze trabecular architecture, L4 vertebrae from each mouse were scanned at a voxel size of 10 µm through a 0.5 mm Al filter (V = 60kV, I = 167µA) with a 0.7-degree angle increment and two frames averaged. Images were reconstructed (nRecon) and rotated (Data Viewer) before calibrating to hydroxyapatite-mimicking phantoms (0.25 and 0.75 g/cm3 Ca-HA). Trabecular bone in the L4 vertebrae was examined over the entire length of the bone.

**S1 Table.** LV4 trabecular properties at 10 and 16 weeks, with p-values from 2-way ANOVA. Significant effects (p>0.05) are highlighted in gray. Significant post-hoc values (p>0.05) between genotypes are marked with *, and between sex are marked with $. Data presented as mean +/- standard deviation.
