# Supplementary figures and images for: Morphological and mechanical characterization of bone phenotypes in the Amish G610C murine model of osteogenesis imperfecta
Source: PLoS One. 2021 Aug 27;16(8):e0255315. doi: 10.1371/journal.pone.0255315 (PMC8396767; doi:10.1371/journal.pone.0255315)

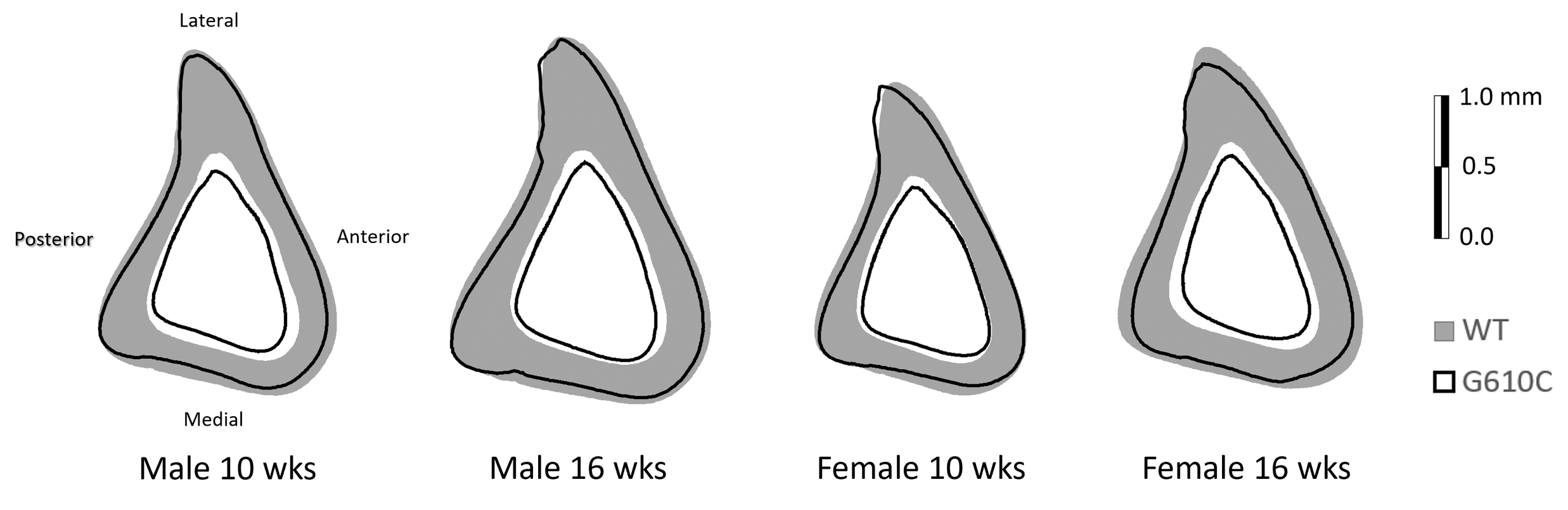

Supplement: S1 Fig — Shown are average profiles of the tibia cortical ROIs from each group, clearly demonstrating that cortical bone area is consistently smaller in G610C mice in both ages and sexes. (TIF) [file pone.0255315.s002.tif]

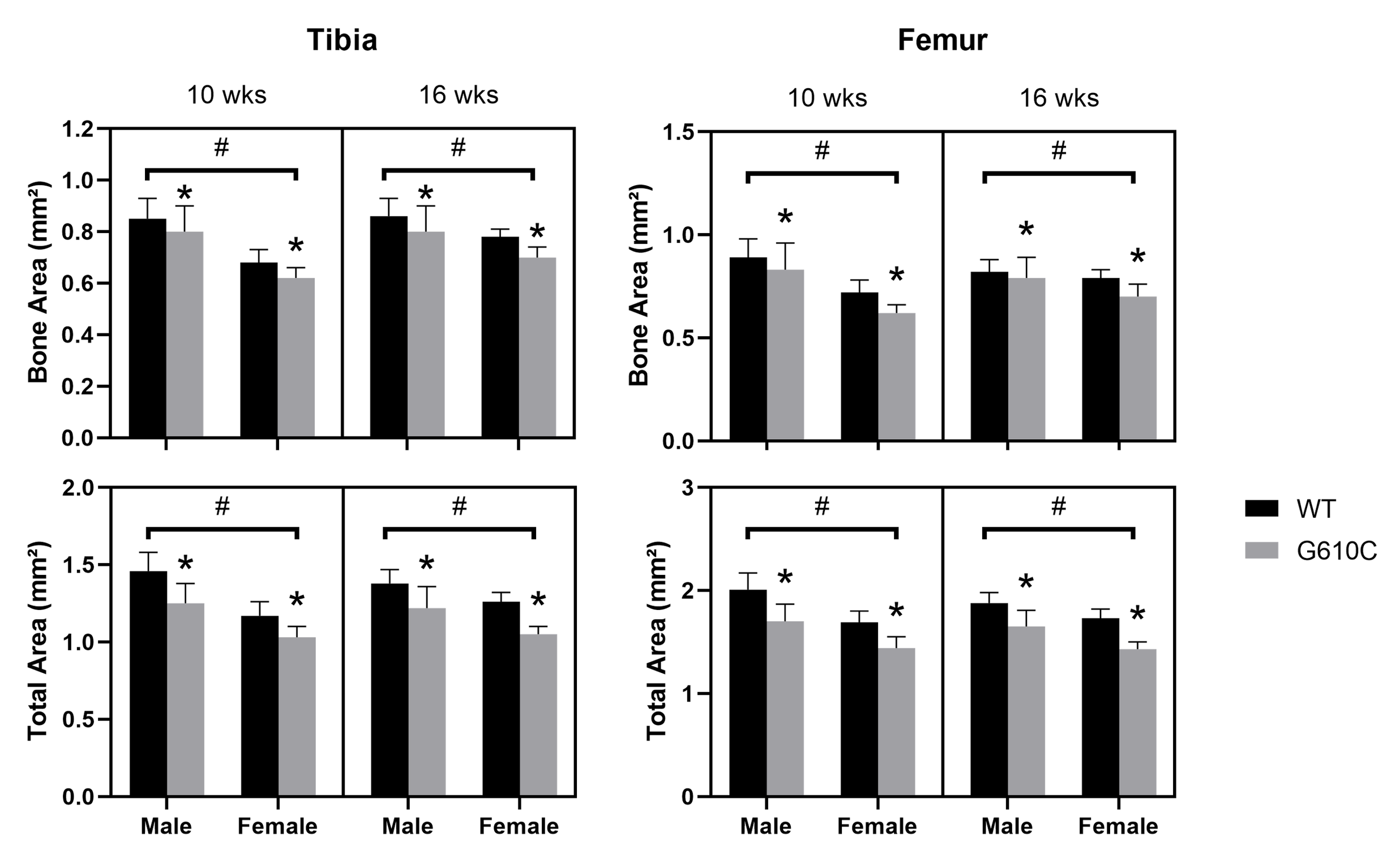

Supplement: S2 Fig — Data shown is mean + standard deviation. Main effects (p-value < 0.05 from 2-way ANOVA) of sex (#) and genotype (*) were seen for all groups, with no interaction. (TIF) [file pone.0255315.s003.tif]

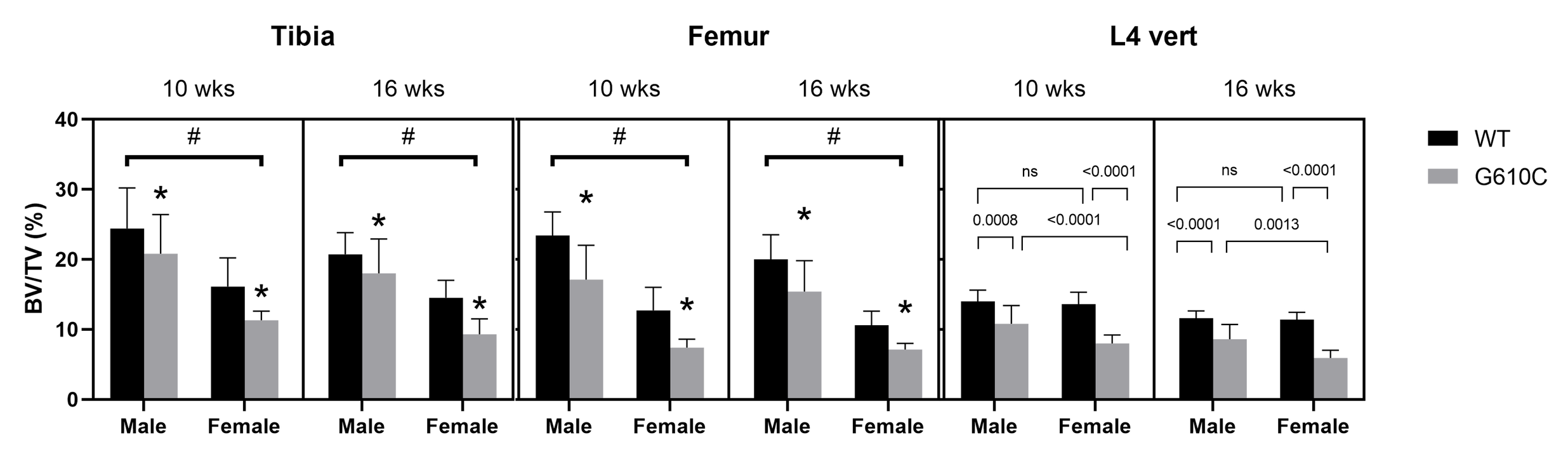

Supplement: S3 Fig — Data shown is mean + standard deviation. Main effects (p-value < 0.05 from 2-way ANOVA) of sex (#) and genotype (*) were seen for all groups. There was a significant interaction effect in only vertebral data; p-values shown are from Tukey post-hoc tests. (TIF) [file pone.0255315.s004.tif]

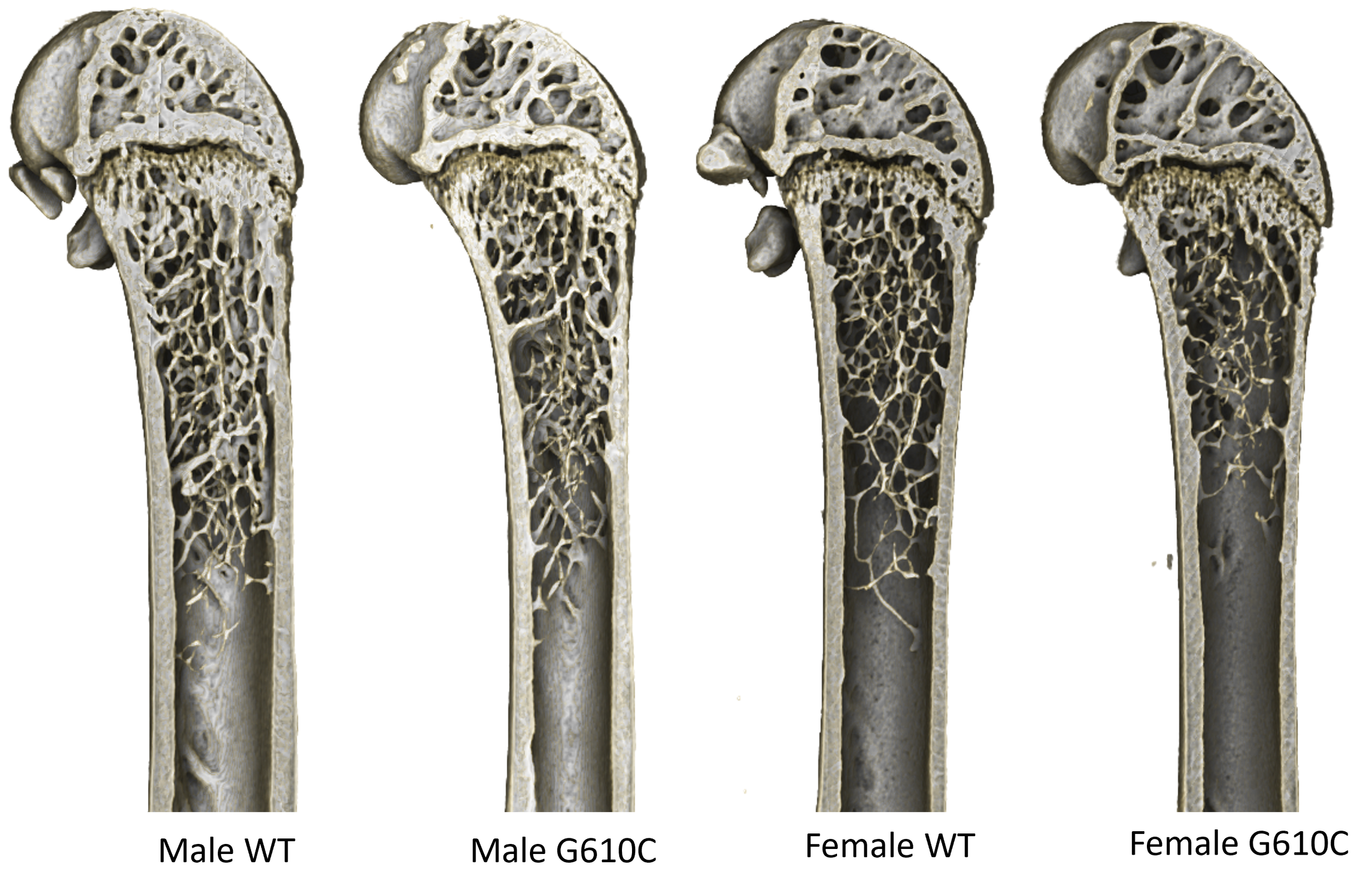

Supplement: S4 Fig — Shown are cross-sectional views of 10-wk femurs from representative mice (the trend is similar in 16-wk mice). Trabecular bone quantity varies significantly between genotypes. (TIF) [file pone.0255315.s005.tif]

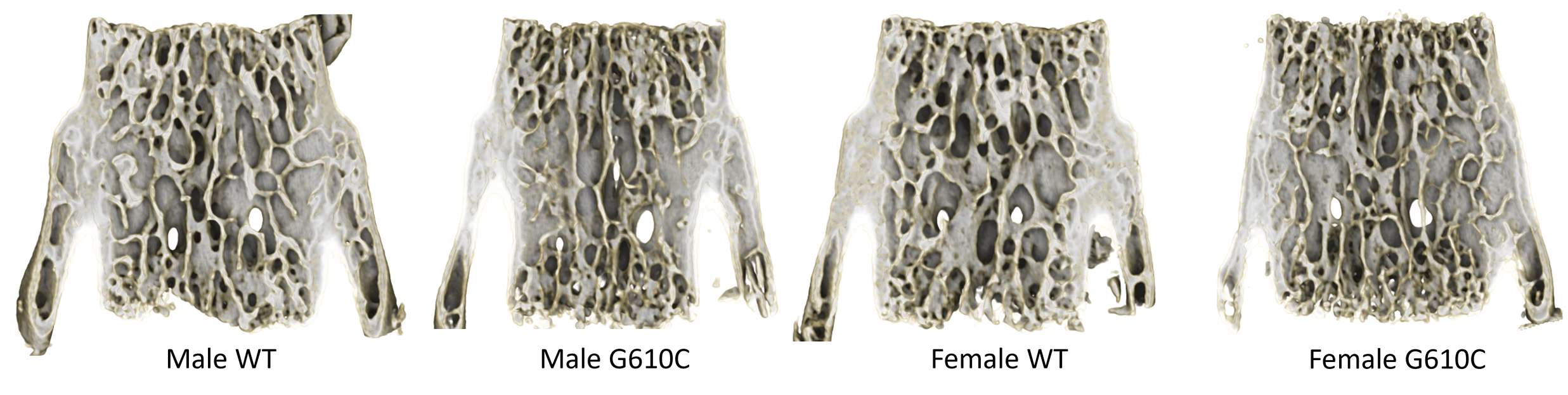

Supplement: S5 Fig — Shown are cross-sectional views of 10-wk L4 vertebrae from representative mice (the trend is similar in 16-wk mice). Trabecular bone quantity varies significantly between genotypes. (TIF) [file pone.0255315.s006.tif]
